# Author Correction: Tyrosine dephosphorylated cortactin downregulates contractility at the epithelial zonula adherens through SRGAP1

**DOI:** 10.1038/s41467-017-02131-w

**Published:** 2017-12-05

**Authors:** Xuan Liang, Srikanth Budnar, Shafali Gupta, Suzie Verma, Siew-Ping Han, Michelle M. Hill, Roger J. Daly, Robert G. Parton, Nicholas A. Hamilton, Guillermo A. Gomez, Alpha S. Yap

**Affiliations:** 1 Division of Cell Biology and Molecular Medicine, The University of Queensland, St. Lucia, QLD 4072 Australia; 2 The University of Queensland Diamantina Institute, Brisbane, QLD 4102 Australia; 3 Cancer Program, Biomedicine Discovery Institute and Department of Biochemistry and Molecular Biology, Monash University, Clayton, VIC 3800 Australia; 4 Program in Membrane Interface Biology, Institute for Molecular Bioscience, The University of Queensland, St. Lucia, QLD 4072 Australia; 5 Centre for Microscopy and Microanalysis, The University of Queensland, St. Lucia, QLD 4072 Australia; 6 Present Address: Centre for Cancer Biology, SA Pathology and the University of South Australia, Adelaide, SA 5000 Australia

*Nature Communications* **8**:790 10.1038/s41467-017-00797-w; Article published online: 5 October 2017

Previous work by Garcia Ponce et al. and Citalán-Madrid et al. suggesting a link between cortactin and RhoA-mediated actomyosin contractility was inadvertently omitted from the original version of this manuscript and should have been cited within the “Discussion” section as follows:

“Previous studies in cortactin-null endothelium and intestinal epithelium revealed hyperactivated RhoA-mediated contractility in these cell types, contrasting with the decrease in junctional RhoA signaling and contractility that we observed, but the direct involvement of cortactin in mediating actomyosin contractility was not established^1,2^. Future research will have to explore the compatibility of these observations.”

1. Garcia Ponce, A. et al. Loss of cortactin causes endothelial barrier dysfunction via disturbed adrenomedullin secretion and actomyosin contractility. *Sci Rep*. **6**, 29003 (2016).

2. Citalán-Madrid, A. F. et al. Cortactin deficiency causes increased RhoA/ROCK1-dependent actomyosin contractility, intestinal epithelial barrier dysfunction, and disproportionately severe DSS-induced colitis. *Mucosal Immunol*. **10**, 1237–1247 (2017).

