# Supplementary material for: Tyrosine dephosphorylated cortactin downregulates contractility at the epithelial zonula adherens through SRGAP1
Source: Nat Commun. 2017 Oct 5;8:790. doi: 10.1038/s41467-017-00797-w (PMC5629210; doi:10.1038/s41467-017-00797-w)
Supplement: Supplementary file 1 — Supplementary Information [file 41467_2017_797_MOESM1_ESM.pdf]

Description of Supplementary Files

File Name: Supplementary Information
Description: Supplementary Figures, Supplementary Tables, Supplementary Methods and Supplementary References

File Name: Supplementary Movie 1
Description: **Supplementary Movie 1**, related to Figure 9a. SRGAP1 KD perturbs HGF stimulated cell motility in Caco-2 cell epithelial monolayer. The cells were transfected with SRGAP1 siRNA (50 nM) 48 hours before HGF treatment (50 ng ml^{-1}, 16 hours). The cells were imaged in RPMI medium plus 2% FBS with or without HGF; .nuclei were identified with NucBlue. Round dots indicate the centres of nuclei. Note the long tracks of dividing cells were not included in further analysis. Movie length, 6 hours. Scale bar = 20 µm.

File Name: Supplementary Movie 2
Description: **Supplementary Movie 2**, related to Figure 9c. SRGAP1 KD perturbs HGF accelerated collective cell migration in Caco-2 cell epithelial monolayer. The cells were transfected with SRGAP1 siRNA (50 nM) 48 hours before HGF treatment (50 ng ml^{-1}, 16 hours). The cells were imaged in RPMI medium plus 2% FBS with or without HGF. Blue colour, nuclei identified with NucBlue. Movie length, 15 hours. Scale bar = 100 µm.

File Name: Peer Review File
Description:

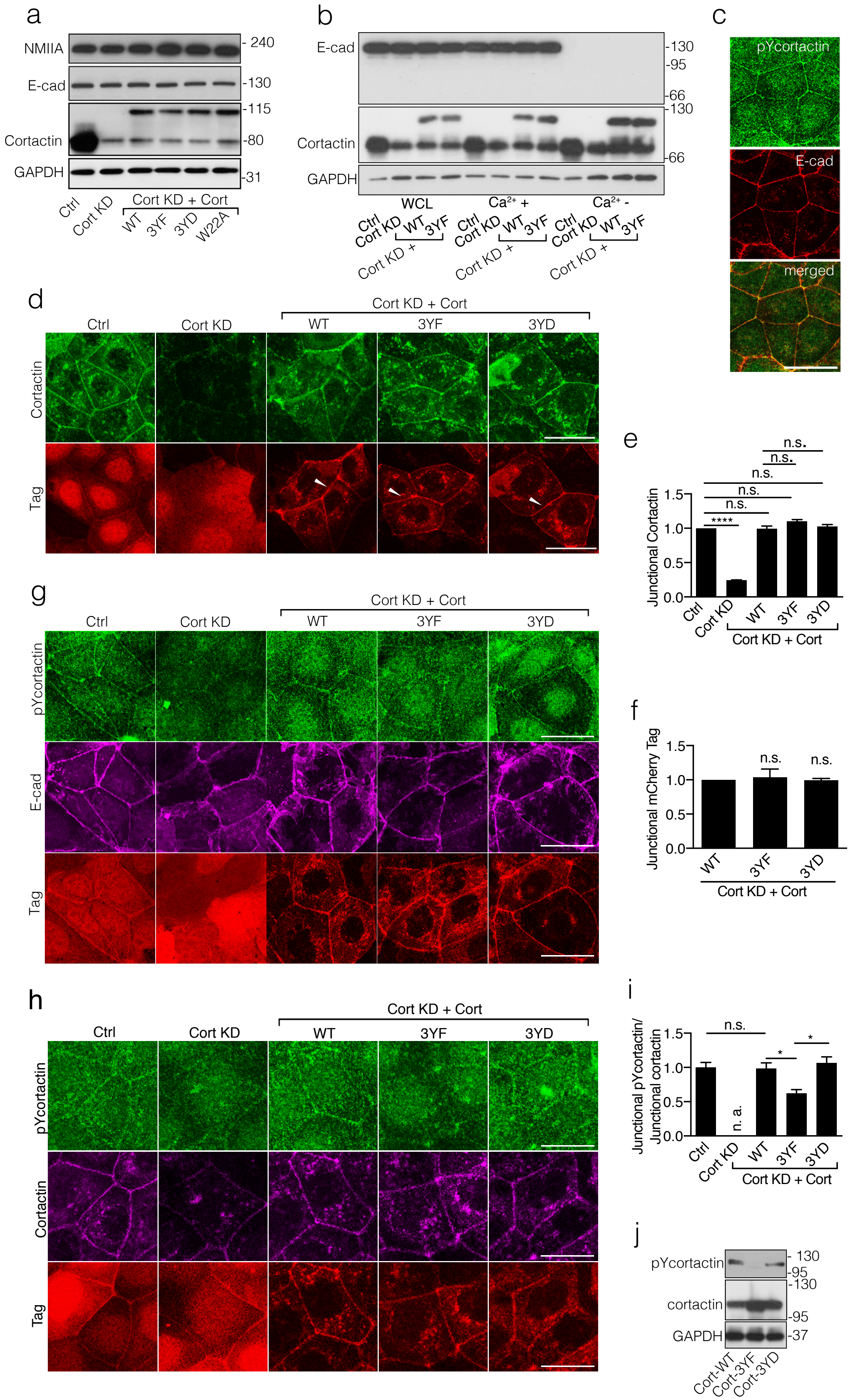

a
NMIIA
E-cad
Cortactin
GAPDH
- 240
-130
-115
-80
-31
Ctrl
Cort KD
Cort KD + Cort
WT
3YF
3YD
W22A
b
E-cad
Cortactin
GAPDH
-130
-95
-66
-130
-66
WCL
Ca2+ +
Ca2+ -
Ctrl
Cort KD
WT
3YF
Cort KD +
c
pYcortactin
E-cad
merged
d
Ctrl
Cort KD
Cort KD + Cort
WT
3YF
3YD
Cortactin
Tag
e
Junctional Cortactin
1.5
1.0
0.5
0.0
****
n.s.
n.s.
n.s.
n.s.
n.s.
Ctrl
Cort KD
WT
3YF
3YD
Cort KD + Cort
f
Junctional mCherry Tag
1.5
1.0
0.5
0.0
n.s.
n.s.
WT
3YF
3YD
Cort KD + Cort
g
Ctrl
Cort KD
Cort KD + Cort
WT
3YF
3YD
pYcortactin
E-cad
Tag
h
Ctrl
Cort KD
Cort KD + Cort
WT
3YF
3YD
pYcortactin
Cortactin
Tag
i
Junctional pYcortactin/
Junctional cortactin
1.5
1.0
0.5
0.0
n.s.
*
*
n. a.
Ctrl
Cort KD
WT
3YF
3YD
Cort KD + Cort
j
pYcortactin
cortactin
GAPDH
- 130
-95
-130
-95
-37
Cort-WT
Cort-3YF
Cort-3YD

Supplementary Figure 1. Cortactin domains differentially regulate contractile cadherin junctions.
Caco-2 cells were transduced with lenti-virus. Asterisks stand for control (Ctrl), cortactin knockdown (Cort KD) or cortactin KD expressing shRNA resistant fluorescent protein-tagged cortactin (WT/3YF/3YD/WT) cells.
(a) Immunoblots of non-muscle myosin IIA (NMIIA), E-cadherin (E-cad), cortactin in cortactin KD and mutants reconstituted Caco-2 cells. GAPDH was used as a loading control. Molecular marker unit, kDa. Overall expression levels of cortactin transgenes are lower than endogenous cortactin as not all KD cells expressed the transgenes.
(b) Surface expression levels of E-cadherin assessed by the trypsin protection assay in Caco-2 cells. Confluent monolayers were trypsinized in Ca^{2+} rich (Ca^{2+}+) or deficient (Ca^{2+}-) HANK's media. WCL, whole cell lysates. The samples were immunoblotted by an antibody against the extracellular domain of E-cadherin. GAPDH was used as a loading control. Molecular marker units, kDa.
(c) Co-immunofluorescence of tyrosine 421-phosphorylated cortactin (pYcortactin) and E-cadherin in Caco-2 cells.
(d) Representative immunofluorescence images of cortactin in Caco-2 cells. (e) Quantification of junctional cortactin. Cortactin-mCherry locates at cell-cell contacts (d, arrow heads) and quantification (f) of junctional cortactin-mCherry.
(g) Representative immunofluorescence images of pYcortactin and E-cad in cortactin KD and reconstitution cells. pYcortactin was labelled by an antibody against pY421-cortactin.
(h) Representative immunofluorescence images of pYcortactin and cortactin in cortactin KD and reconstitution cells.
(i) Quantification of junctional pYcortactin from experiments of (g) normalized to junctional cortactin. n.a., not applicable.
(j) Immunoblots of pYcortactin and cortactin in WT/3YF/3YD-cortactin-mCherry overexpressed Caco-2 cells. Molecular marker units, kDa.
N = 3 independent experiments, data are means ± s.e.m.; one-way ANOVA with Dunnett's post hoc analysis; n.s., not significant; *, $p<0.05$; ****, $p<0.0001$. Scale bars = 25 µm.

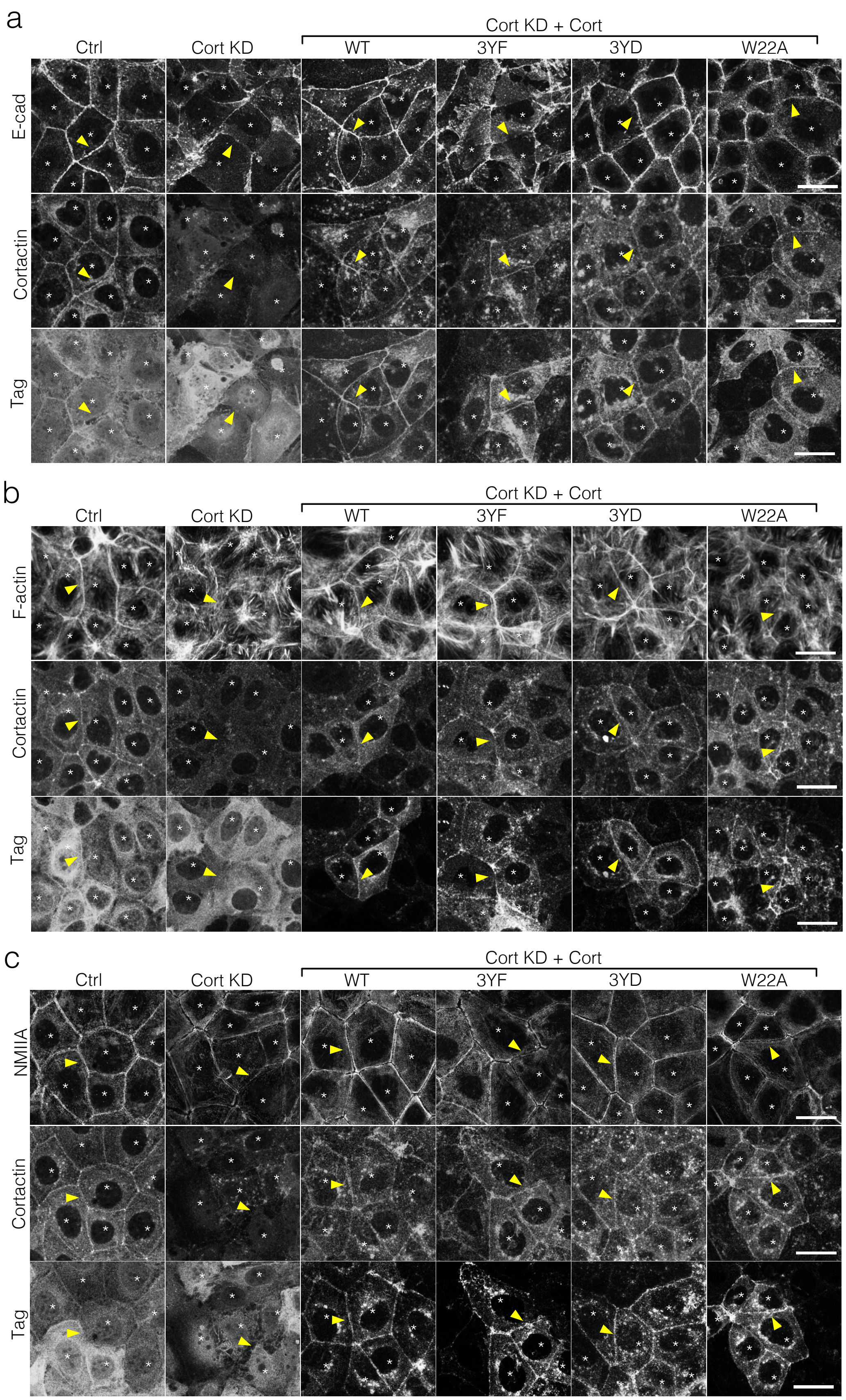

Supplementary Figure 2. Confocal images of cortactin and fluorescent protein tags corresponding to the images of Figure 1c.
Immunofluorescence costaining of cortactin and fluorescent protein tags with E-cadherin (E-cad, a), F-actin (b) and non-muscle myosin IIA (NMIIA, c). The images are maximum projections of the three apical-most sections. Transduced cells are indicated with asterisks. Arrowheads indicate homotypic junctions. Scale bars = 25 µm.

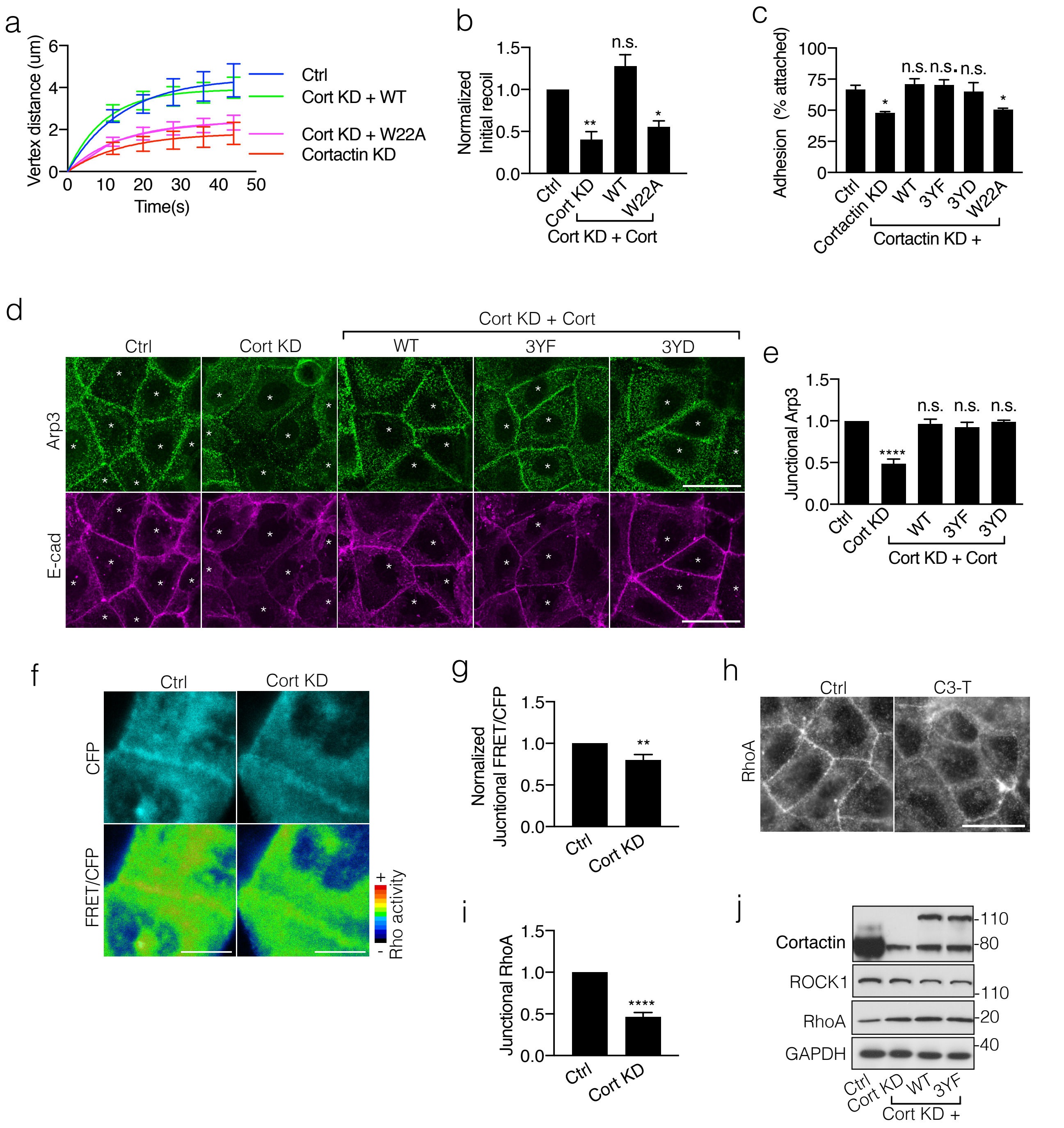

Supplementary Figure 3. Cortactin tyrosine-phosphorylation regulates junctional Arp2/3 and RhoA.
(a and b) Cortactin interaction with Arp2/3 is essential for junctional contractility. Records of vertices separation (a, plots = means ± s.e.m of 3 independent experiments, at least 12 junctions were measured in each experiment) and normalized initial recoil (b) after laser ablation in control (ctrl), cortactin knockdown (cort KD) and cortactin mutants (WT/W22A) reconstituted Caco-2 cells. N = 3 independent experiments, data are means ± s.e.m.; one-way ANOVA with Dunnett's post hoc analysis; n.s., not significant; *, p<0.05; **, p<0.01.
(c) Cell-substrate (fibronectin) of control, cortactin KD and KD cells reconstituted with cortactin mutants. N = 4 independent experiments, data are means ± s.e.m.; one-way ANOVA with Dunnett's post hoc analysis; n.s., not significant; *, p<0.05.
(d,e) Cortactin tyrosine phosphorylation does not affect junctional Arp2/3. Representative immunofluorescence images (d) of Arp3 and E-cadherin (E-cad), and quantification (e) of junctional Arp3 in Caco-2 cells. Asterisks stand for lentivirus transduced cells. N = 3 independent experiments, data are means ± s.e.m.; one-way ANOVA with Dunnett's post hoc analysis; n.s., not significant; ****, p<0.0001. Scale bars = 25 µm.

(f) Representative images of RhoA activity in Caco-2 cells. RhoA activity was measured by a RhoA FRET biosensor. Scale bars = 10 µm. (g) Quantifications of junctional FRET normalized to biosensor expression (FRET/CFP) signals from experiments of (f). N = 3 independent experiments, data are means ± s.e.m.; Student's t-test; **, $p<0.01$.
(h,i) Immunofluorescence images of RhoA (h) and quantification of junctional RhoA (i) in control and C3 transferase (C3-T; 200 ng ml^{-1}, 2 hours) treated Caco-2 cells. N = 3 independent experiments, data are means ± s.e.m.; Student's t-test; ****, $p<0.0001$.
(j) Immunoblots of cortactin, ROCKI and RhoA in control, cortactin KD and cortactin mutants (WT/3YF) reconstituted Caco-2 cells. GAPDH was used as a loading control. Molecular marker units, kDa.

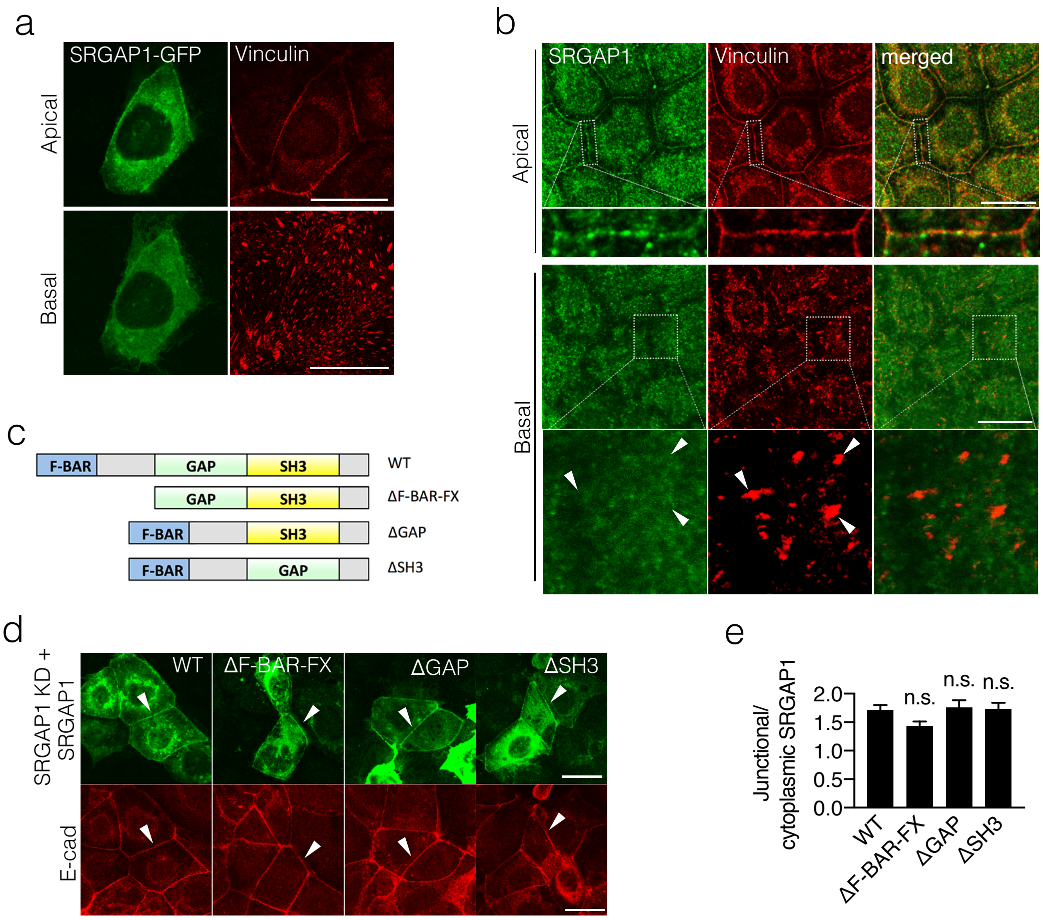

Supplementary Figure 4. Localization of SRGAP1 in Caco-2 cells.
(a) Localization of SRGAP1-GFP and vinculin at the apical region and the basal region of Caco-2 cells.
(b) Dual-colour immunofluorescence of SRGAP1 and vinculin at the apical region and the basal region of Caco-2 cells. Arrowheads, SRGAP1 shows no visible co-staining with vinculin.
(c-e) Domain deletion analysis of junctional SRGAP1 localization. (c) Schematic of SRGAP1 domains and domain-depletion mutants. Representative images (d) of the localization of SRGAP1 mutants in Caco-2 cells and quantification (e) of junctional SRGAP1 normalised to cytoplasmic SRGAP1. SRGAP1 was labelled by an antibody against myc-tag. Arrowheads, SRGAP1 at cell-cell contacts. n = 30-45 junctions from one experiment (representative of 3 independent experiments), data are means ± s.e.m.; one-way ANOVA with Dunnett's post hoc analysis; n.s., not significant. Scale bars = 25 µm.

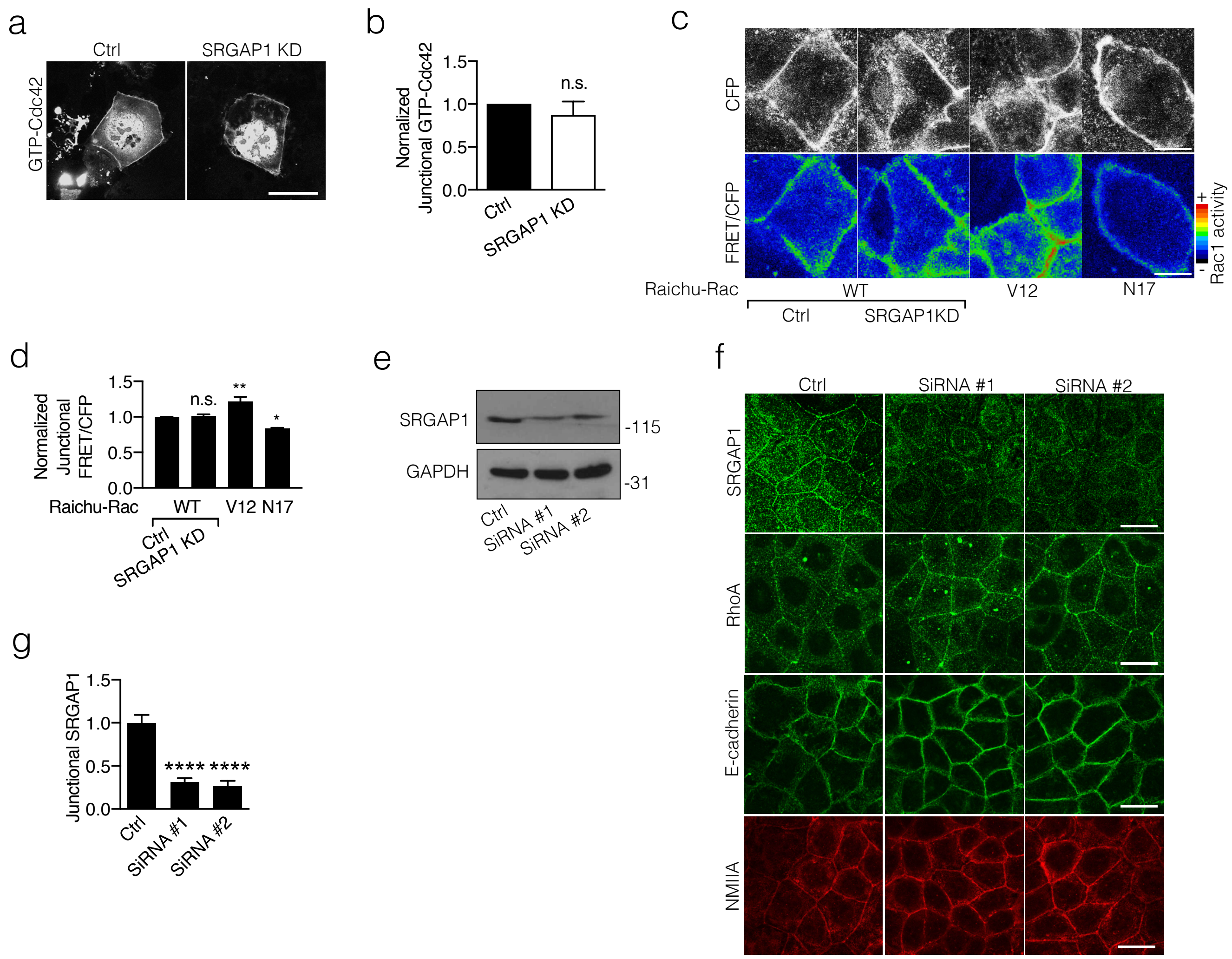

Supplementary Figure 5. SRGAP1 regulates junctional RhoA signalling.
(a and b) SRGAP1 is not a Cdc42 GAP at the junctions.
(a) Representative live cell images of YFP-CRIB that monitors GTP Cdc42 in control (ctrl) and SRGAP1 knockdown (KD) Caco-2 cells. Scale bar = 25 µm. (b) Quantifications of junctional GTP Cdc42 normalized to cytoplasmic fluorescence intensities.
(c and d) SRGAP1 is not a Rac1 GAP at the junctions.
(c) Representative images of Rac1 activity in Caco-2 cells. Junctional Rac1 activity was measured by WT/V12/N17-Raichu-Rac FRET biosensors. Scale bars = 10 µm. (d) Quantifications of junctional FRET normalized to biosensor expression (FRET/CFP) signals from experiments of (c). N = 3 independent experiments, data are means ± s.e.m.; one-way ANOVA with Dunnett's post hoc analysis; n.s., not significant; *, $p<0.05$; **, $p<0.01$.
(e - g) Caco-2 cells were transfected with a control siRNA, SRGAP1 siRNA#1 or siRNA#2. (e) Immunoblots of SRGAP1 in control and SRGAP1 KD cells. (f) Representative immunofluorescence images of SRGAP1, RhoA, E-cadherin and NMIIA in control and SRGAP1 KD cells. Scale bars = 25 µm. (g) Quantifications of junctional SRGAP1 from the experiments of (f). N = 25-30 junctions from one representative experiment, data are means ± s.e.m.; one-way ANOVA with Dunnett's post hoc analysis; ****, $p<0.0001$.

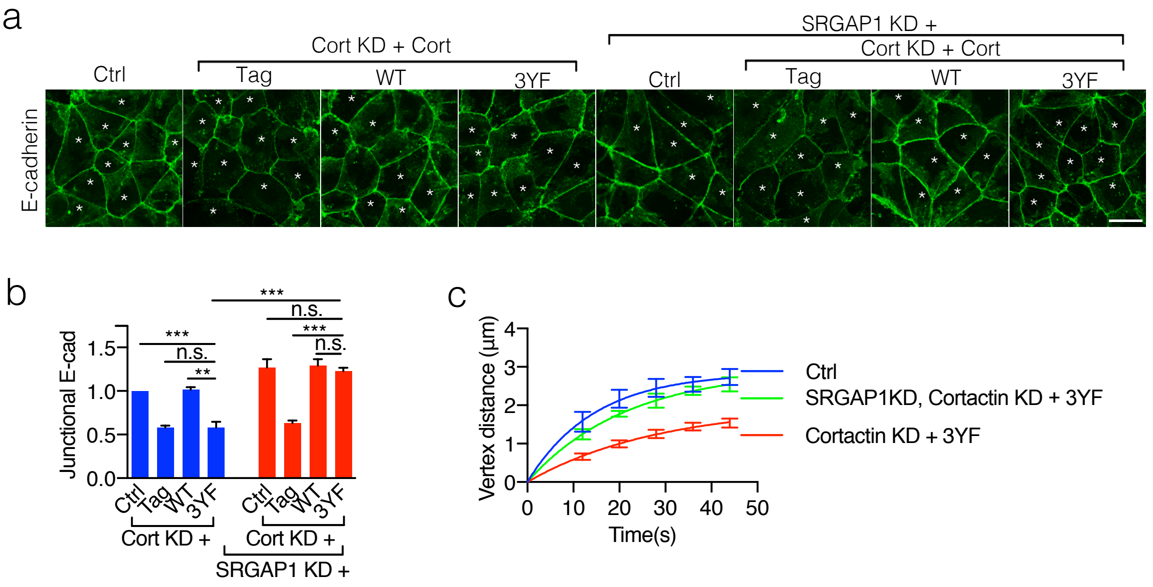

Supplementary Figure 6. Cortactin tyrosine dephosphorylation downregulates E-cadherin junctions via SRGAP1.

Control (ctrl) Caco-2 expressing mCherry or Caco-2 cells where cortactin was depleted by shRNA (cort KD) and mCherry-tagged shRNA resistant cortactin variant (cort KD + WT/3YF/3YD) was expressed are indicated with asterisks in images. In these experiments SRGAP1 was depleted by siRNA transfection.

(a) Representative images of apical E-cadherin in Caco-2 cells. Scale bar = 25 µm.

(b) Quantification of junctional E-cadherin. N = 3 independent experiments, data are means ± s.e.m.; one-way ANOVA with Dunnett's post hoc analysis; n.s., not significant; **, $p<0.01$; ***, $p<0.001$.

(c) Records of vertices separation after laser ablation in control, 3YF reconstituted, or SRGAP1 KD and 3YF reconstituted Caco-2 cells. Plots = means ± s.e.m. of 3 independent experiments, at least 12 junctions were measured in each experiment.

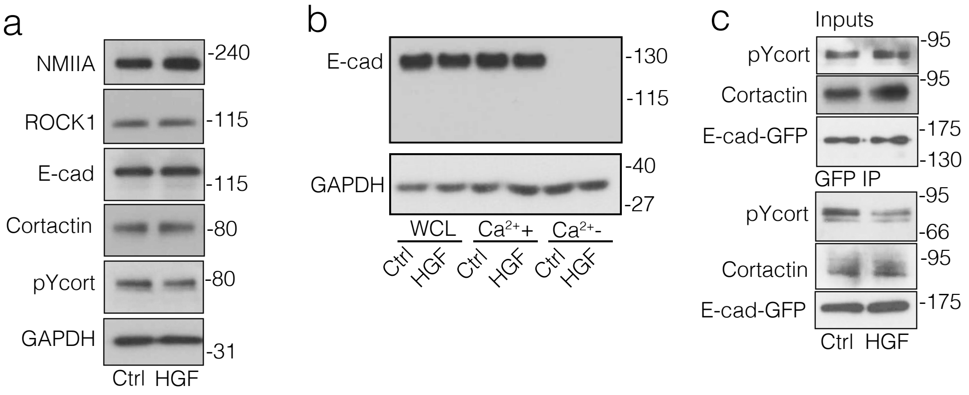

Supplementary Figure 7. Effects of HGF on the ZA.
(a) Immunoblots against NMIIA, E-cadherin (E-cad), cortactin, tyrosine 421-phosphorylated cortactin (pYcort) in control (ctrl) or HGF treated Caco-2 cells. GAPDH was used as a loading control.
(b) Surface expression levels of E-cadherin in control or HGF treated Caco-2 cells. Confluent monolayers were trypsinized in Ca^{2+} rich (Ca^{2+}+) or deficient (Ca^{2+}-) HANK's media. WCL, whole cell lysates. The samples were immunoblotted by an antibody against the extracellular domain of E-cadherin.
(c) Immunoblots of GFP-trap precipitations from E-cadherin-GFP expressed control and HGF treated Caco-2 cells. Molecular marker units, kDa.

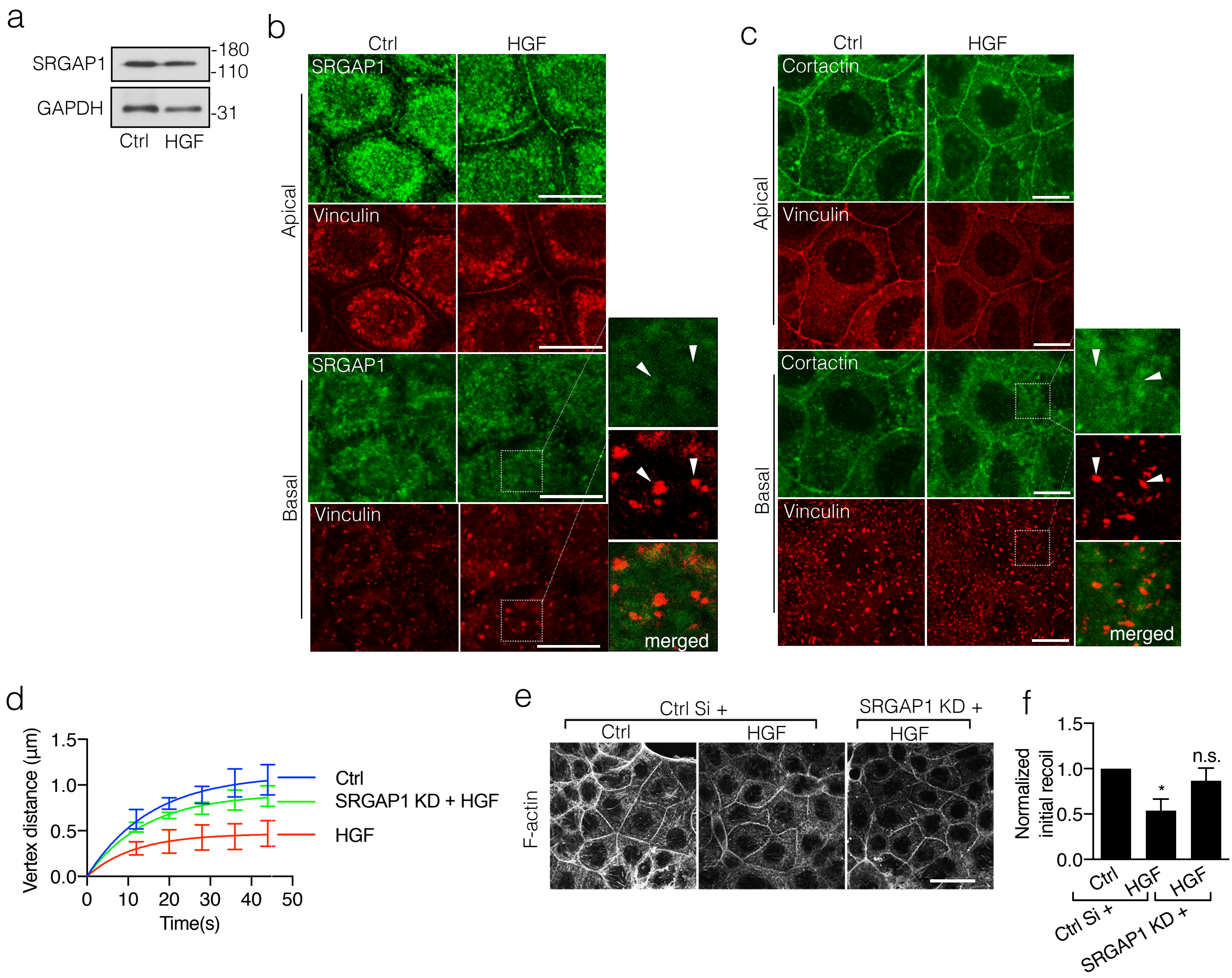

Supplementary Figure 8. Effects of HGF in Caco-2 cells.
(a) Immunoblots of SRGAP1 in control (ctrl) and HGF treated Caco-2 cells. GAPDH was served as a loading control. Molecular marker units, kDa.
(b and c) Immunofluorescence confocal images of SRGAP1 (b) and cortactin (c) at the apical and basal regions of control and HGF treated Caco-2 cells. Arrowheads indicate cell-matrix contacts labelled by vinculin where no obvious SRGAP1 or cortactin signal is observed.
(d) Records of vertices distances after laser ablation in control, HGF treated or HGF treated SRGAP1 KD Caco-2 cells. Plots = means ± s.e.m. of 3 independent experiments, at least 12 junctions were measured in each experiment.
(e and f) Representative confocal images (e) and quantification (f) of apical F-actin in control and SRGAP1 KD cell monolayers treated (HGF) or not with HGF. N = 3 independent experiments, data are means ± s.e.m.; one-way ANOVA with Dunnett's post hoc analysis; *, $p<0.05$; **, $p<0.01$. Scale bars = 15 µm.

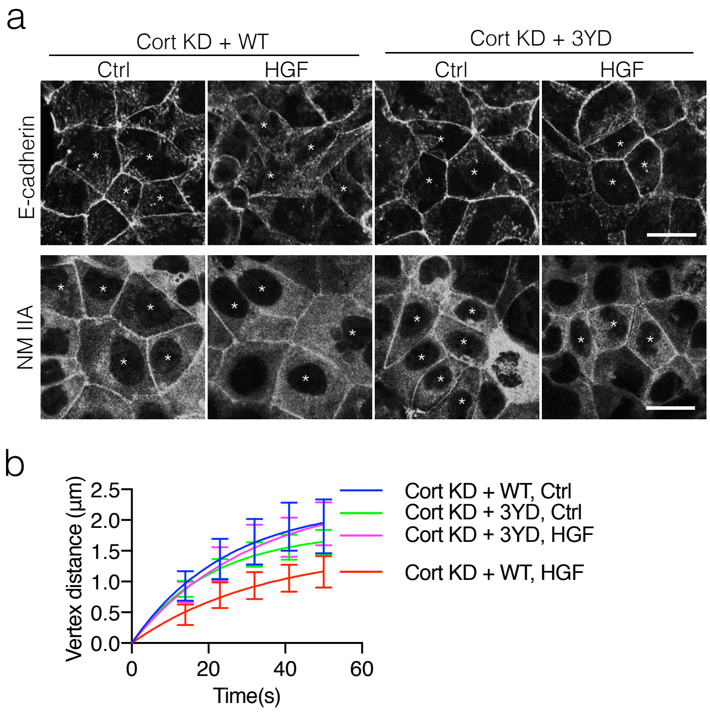

Supplementary Figure 9. HGF disrupts the contractile ZA via SRGAP1.
Cortactin knockdown (cort KD) Caco-2 cells were reconstituted with mCherry-tagged WT or 3YD cortactin are indicated by the asterisks.
(a) Representative confocal images of E-cadherin and non-muscle myosin IIA (NMIIA) in the Caco-2 cells upon HGF.
(b) Records of vertices separation after laser ablation in the Caco-2 cells with or without HGF. Plots = means ± s.e.m. of 3 independent experiments, at least 12 junctions were measured in each experiment.

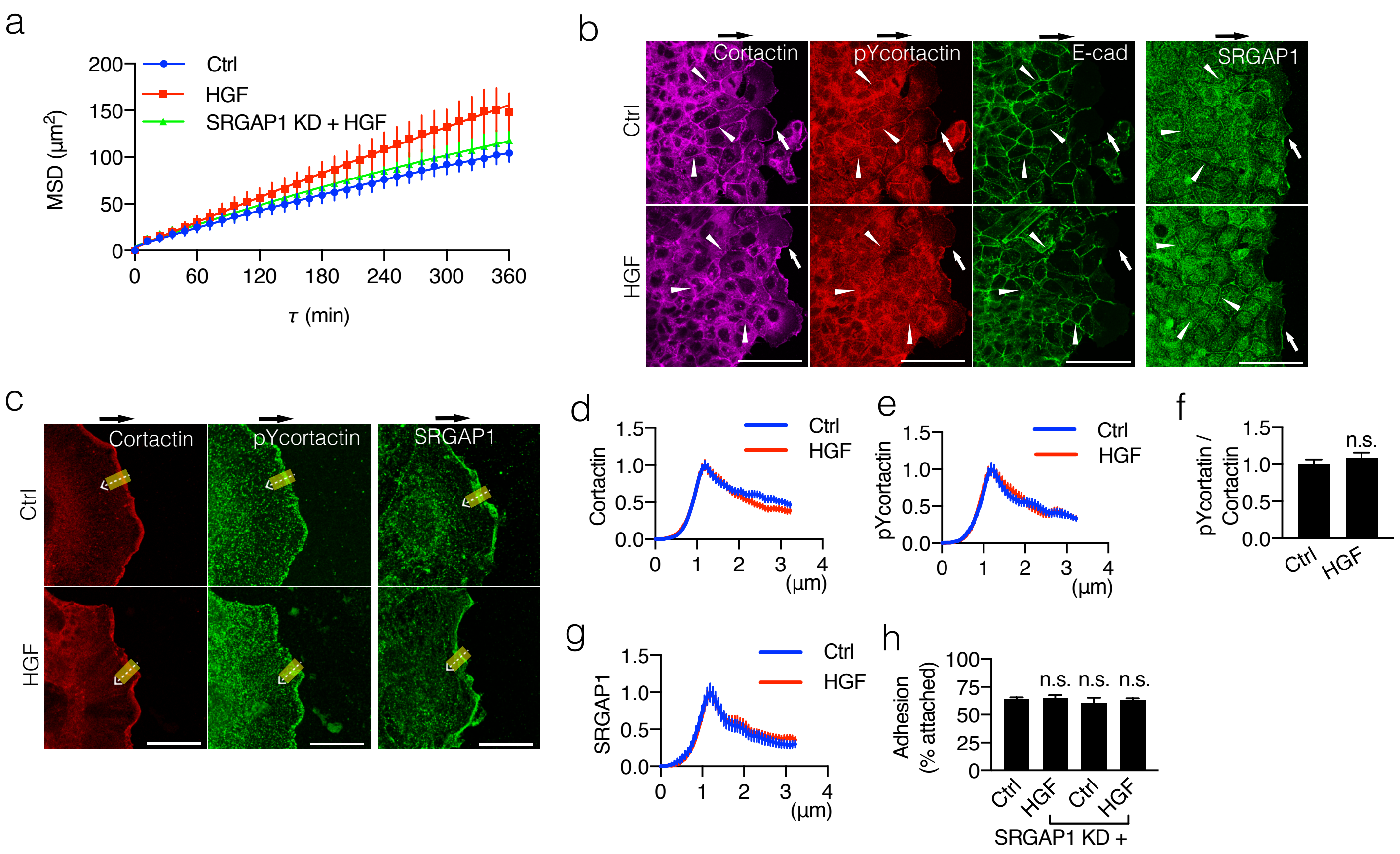

Supplementary Figure 10. HGF promotes the movement of Caco-2 monolayers.
(a) The mean squared displacement (MSD) of the moving nuclei in confluent Caco-2 monolayers. Plots = means ± s.e.m. of 3 independent experiments, 750 - 1000 nuclear tracks were analysed in each experiment.
(b) Representative Z-axis maximum value projected confocal images of the migrating epithelia at 8-hour post the start of migration. White arrowheads indicate cell-cell contacts; white arrows indicate lamellipodia. HGF reduced tyrosine 421-phosphorylated (pY) cortactin and increased SRGAP1 at cell-cell contacts of the migrating monolayers. Scale bars = 50 μm. Black arrows indicate the direction of migration.
(c - g) HGF did not change cortactin, pYcortactin or SRGAP1 at the migration edge.
(c) Representative immunofluorescence images (c) of the cell periphery at the migration edge of the epithelium. Scale bars = 10 μm. Black arrows indicate the direction of migration. Plotted line scans of cortactin (d), pYcortactin (e) and SRGAP1 (g) at the cell peripheries that are exemplified by the yellow blocks in (c). Dashed arrows indicate direction of the analysis of (d-g). (f) Ratio of pYcortactin immunofluorescence peak values over cortactin peak values at the migration edge. N = 51(cortactin and pYcortactin), 54 (SRGAP1) cells, data are means ± s.e.m.; students' t-test; n.s., not significant.
(h) Cell-substrate (fibronectin) adhesion of control, SRGAP1 KD and HGF treated Caco-2 cells after detachment. SRGAP1 siRNA (100 nM) was transfected 48 hours before the experiment. HGF (50 ng ml-1, in RPMI containing 2% FBS) was added 13 hours before the experiment.

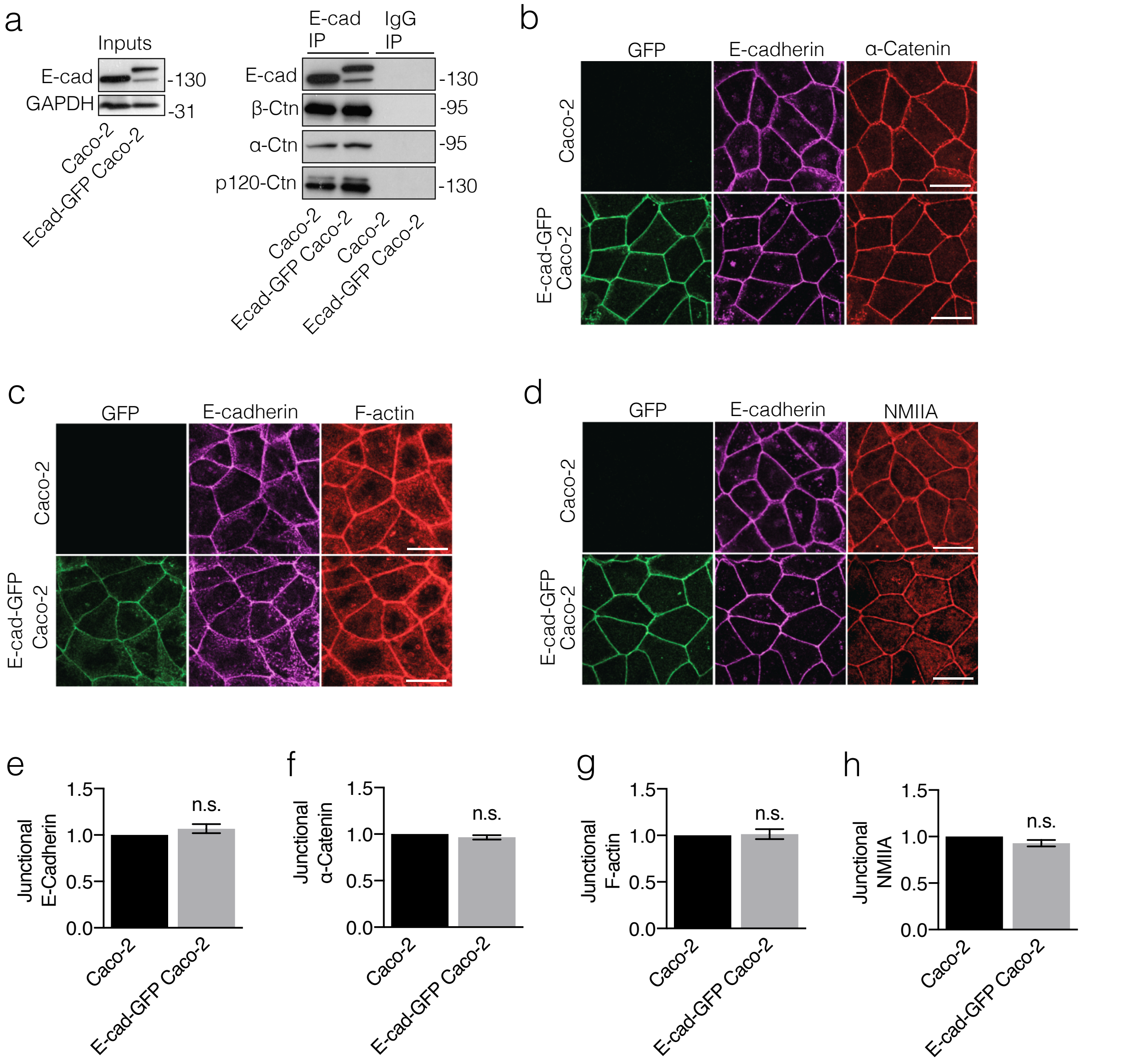

Supplementary Figure 11. Characterization of genome-edited E-Cad-GFP cells.
(a) E-cadherin (E-cad) was immunoprecipitated from Caco-2 cells and Caco2 E-cad-GFP cells (B1C2) and immunoblotted for E-cadherin, β-catenin (β-Ctn), α-catenin (α-Ctn) and p120 catenin (p120-Ctn). Input lysates were immunoblotted for E-cadherin and GAPDH, which served as control for loading.
(b-h) Representative images and fluorescence intensity quantification of junctional E-Cadherin (b,e), α-catenin (b,f), NMIIA (c,g) and F-actin (d,h) in Caco-2 and E-cad-GFP Caco-2 cells. N = three experiments, data are means ± s.e.m.; students' t-test; n.s., not significant. Scale bars = 20 µm.

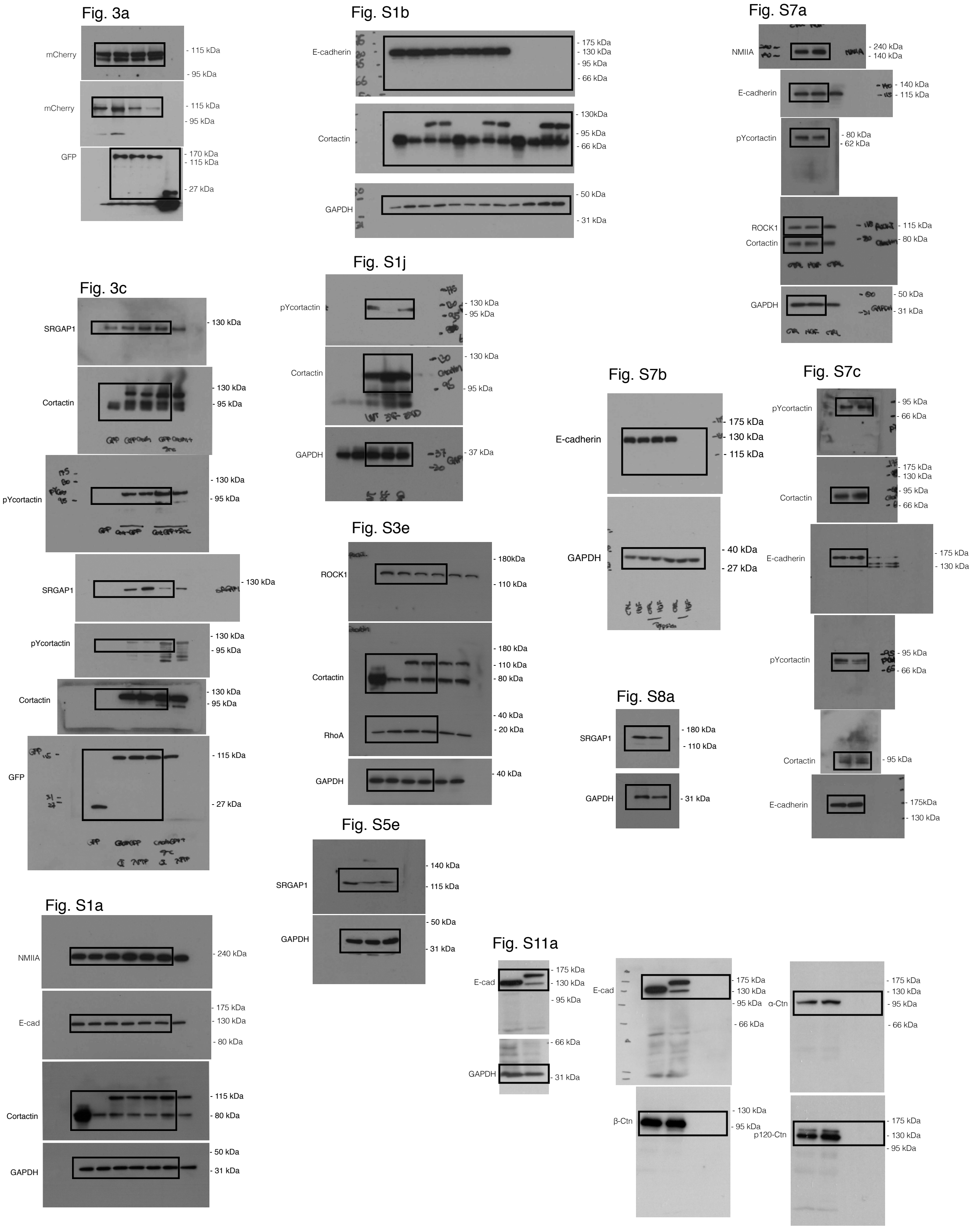

Supplementary Figure 12. Uncropped Western blots.

Treatment	K values	P<0.05
Control<br>Cortactin KD<br>Cortactin KD + WT<br>Cortactin KD + 3YF<br>Cortactin KD + 3YD<br>(Fig. 1a,b)	0.075 ± 0.004, n=3<br>0.075 ± 0.003, n=3<br>0.064 ± 0.001, n=3<br>0.064 ± 0.009, n=3<br>0.067 ± 0.003, n=3	n.s.
Control<br>SRGAP1 KD<br>(Figure 4f,g)	0.065 ± 0.008, n=3<br>0.080 ± 0.002, n=3	n.s.
Cortactin KD + WT<br>Cortactin KD + 3YF<br>SRGAP1 KD + Cortactin KD + 3YF<br>(Fig. 5e, S6c)	0.053 ± 0.008, n=3<br>0.049 ± 0.023, n=3<br>0.057 ± 0.010, n=3	n.s.
Control<br>HGF<br>(Fig. 6c,d)	0.064 ± 0.012, n=3<br>0.059 ± 0.015, n=3	n.s.
Control<br>HGF<br>SRGAP1 KD + HGF<br>(Fig. 7g, S8d)	0.080 ± 0.019, n=3<br>0.085 ± 0.014, n=3<br>0.064 ± 0.007, n=3	n.s.
Cortactin KD + WT, Control<br>Cortactin KD + WT, HGF<br>Cortactin KD + 3YD, Control<br>Cortactin KD + 3YD, HGF<br>(Fig. 8f, S9b)	0.062 ± 0.011, n=3<br>0.035 ± 0.007, n=3<br>0.058 ± 0.006, n=3<br>0.064 ± 0.016, n=3	n.s.

Supplementary Table 1, related to Figure 1, 4-8. The k values obtained from non-linear fitting of junctional recoil measurements after laser ablation. Data are means of ± SEM; one-way ANOVA with Dunnett's post hoc test; n.s., not significant, p>0.05.

Relation/kinetic equation	a	b	K	Eq
1. RhoA→ROCK1 (stimulation) $\frac{dROCK1}{dt} = -a_1 ROCK1 + b_1 \theta_{RhoA}^{ROCK1}$	a_1=0.15†	b_1=0.2	$K_{RhoA}^{ROCK1} = 0.5$	(Eq. 1)
2. ROCK1—I Rnd3 (repression) $\frac{dRnd3}{dt} = -a_2 Rnd3 + b_2 (1 - \theta_{ROCK1}^{Rnd3})$	a_2=0.07	b_2=0.2	$K_{ROCK1}^{Rnd3} = 0.5$	(Eq. 2)
3. Rnd3→p190B (stimulation) $\frac{dp190B}{dt} = -a_3 p190B + b_3 \theta_{Rnd3}^{p190B}$	a_3=0.1	b_3=0.3	$K_{Rnd3}^{p190B} = 0.5$	(Eq. 3)
4. p190B—I RhoA (repression) $\frac{dRhoA}{dt} = -a_4 RhoA + b_4 (1 - \theta_{p190B}^{RhoA})$	a_4=0.1††	b_4=0.3	$K_{p190B}^{RhoA} = 0.5$	(Eq. 4)
5. SRGAP1—I RhoA (repression) $\frac{dRhoA}{dt} = -a_4 RhoA + b_5 (1 - \theta_{SRGAP1}^{RhoA})$	a_4=0.1††	b_5=0.1	$K_{SRGAP1}^{RhoA} = 0.1$	(Eq. 5)
6. ROCK1→NMIIA (stimulation) $\frac{dNMIIA}{dt} = -a_5 NMIIA + b_5 \theta_{ROCK1}^{NMIIA}$	a_6=0.1	b_6=0.2	$K_{ROCK1}^{NMIIA} = 0.5$	(Eq. 6)
7. NMIIA→ROCK1 (stimulation) $\frac{dROCK1}{dt} = -a_1 ROCK1 + b_6 \theta_{NMIIA}^{ROCK1}$	a_1=0.15††	b_7=0.2	$K_{NMIIA}^{ROCK1} = 3$	(Eq. 7)
† An Hill coefficient of n=2 was used throughout all modelling [1]. In all simulations, initial junctional concentrations were set to 1 unless otherwise stated. ††Note that a_1 and a_4 correspond to a single decay factor for ROCK1 and RhoA, respectively.				

Supplementary Table 2, related to Figure 5f, g. System of differential equations and the Hill (K), maximal association rates (b) and decay (a) constants in the stimulation-repression model of the network shown in Fig. 5f †.

Supplementary Methods

Generation and characterization of E-Cad-GFP Caco-2 cells

Caco-2 cells were transfected with gRNA targeting the last exon of E-cadherin gene (GTTCAAGTAGTCATAGTCCTGG), Cas9 (Addgene plasmid #48139) and the donor construct consisting of GFP flanked by homologous sequence of the human E-cadherin gene. The GFP positive cells were sorted and seeded at low density on a culture dish 48 hours post transfection. Isolated cell colonies were sub-cultured and characterized for insertion of GFP by immunoblotting and sequencing the genomic DNA. One of the Caco2 clones (B1C2) was further characterized. E-Cadherin-GFP co-immunoprecipitated β-catenin, α-catenin, and p120-catenin to the same extent as did E-Cadherin from a control Caco-2 line (Supplementary Fig 11a). Furthermore, quantitative fluorescence microscopy showed identical levels of E-cadherin (Supplementary Fig b,e), α-catenin (Supplementary Fig. 11b,f), NMIIA (Supplementary Fig. 11c,g) and F-actin (Supplementary Fig. 11d,h) in the E-Cad-GFP Caco-2 line as in control Caco-2 cells.

Live cell imaging

For live cell imaging, nano-ablation, FRAP and FRET experiments, the cells were grown in 29 mm diameter borosilicate glass-bottomed dishes (Shengyou Biotechnology) and imaged in movie medium (15 mM HEPES, pH=7.5; 5 mM $CaCl_2$; 10 mM D-glucose; 2% FBS in Hank's balance salt solution) at 37 °C. For nuclei tracking and migration assay experiments, the cells were grown in borosilicate glass-bottomed chambers (Lab-Tek) and imaged in RPMI medium supplied with 2% FBS at 37 °C and 5% CO_2.

GFP-trap precipitation [1]

The GFP-trap precipitation was performed with Caco-2 or HEK-293T cells. The cells were plated into 15 cm cell culture dishes and growth to 50% confluence for transfection with plasmids encoding GFP tagged proteins using lipofectamine 2000 (Invitrogen). 30 µg of plasmids were transfected into each 15 cm dish and three dishes were combined for each group of the experiments. 48 hours post transfection, the cells were harvested and lysed with 0.5% NP-40 in dilution buffer (10 mM Tris-HCl, pH 7.5; 150 mM NaCl; 0.5 mM EDTA) 30 min on ice. After 12,500 g, 10 min centrifugation, the supernatants were collected. 5% of the lysates were stocked as inputs. The rests of the lysates were added to 60 µl of GFP-Trap agarose beads (Sigma) and incubated at 4 °C overnight with rotation. Beads were then washed (10 mM Tris-HCl, pH 7.5; 300 mM NaCl; 0.5 mM EDTA) three times on ice to remove non-specific binding, boiled in the Western blotting lysis buffer and then centrifuged at 12,500 g; 10 min. The supernatants were kept as protein samples subjected to SDS-PAGE and Western blotting.

Interaction screen

The interaction screen was performed based on mass spectrometric analysis. To identify proteins whose interaction with cortactin might be influenced by its tyrosine-phosphorylation status, the WT cortactin and 3YF cortactin cDNAs were cloned into the Pc-Bio vector [2]. The plasmids were then co-transfected with pUASp-BirA plasmid into Caco-2 cells and the cells were cultured in 50% RPMI/50% F10 mixed medium with 10% FBS. 48 hours post transfection, the cells were washed and scraped into wash buffer (50 mM HEPES, pH 7.4; 150 mM NaCl) on ice, pelleted and lysed (50 mM HEPES, pH 7.4; 150 mM NaCl; 0.5% Triton X-100, with phosphatase inhibitor cocktail; 30 min on ice with agitation). Supernatants were collected after centrifugation (12,500 g, 10 min) and incubated with streptavidin-coated Dynabeads (M280, Invitrogen, 50 µl, pre-blocked with 0.1% BSA, 4 °C overnight with rotation). Beads were then collected, washed with dilution buffer (50 mM HEPES, pH 7.4; 300 mM NaCl), boiled at 100 °C 10 min in Western blotting lysis buffer to release the proteins and the supernatants were collected after centrifugation (12,500 g, 10 min) for mass spectroscopy. Peptides were identified by GeLC-MS/MS with a semi-automated procedure [3] and LC-MS/MS and database searching were performed [4]. Bound proteins identified in the Pc-Bio vector expression group were considered as background. The scores of proteins shown in each profile were normalized to the score of cortactin in the corresponding profile. The resulting values were compared between the WT and 3YF cortactin profiles.

In vitro tyrosine dephosphorylation assay

HEK-293T cells were transfected with Cortactin-GFP (pEGFP-N1 backbone) plasmid plus SRGAP1 siRNA (50 nM), with or without Src^{Y527F}-mCherry (pmCherry-N1 backbone) plasmid. The cells were lysed 48 hours post transfection (10 mM Tris-HCl, pH 7.5; 150 mM NaCl; 0.5 mM EDTA; 0.5% NP-40, protease inhibitors [Roche], phosphatase inhibitor cocktail [Invitrogen]), and the lysates were subjected to GFP-trap precipitation. After overnight incubation at 4 °C with GFP-trap beads, the beads were washed thoroughly with wash buffer (10 mM Tris-HCl, pH 7.5; 300 mM NaCl). For in vitro tyrosine dephosphorylation, the beads were incubated with 6,000 units Lambda protein phosphatase (New England BioLabs, Cat#P0753) in 300 µl

protein phosphatase reaction buffer (50 mM HEPES, pH=7.5; 1 mM $MgCl_2$; 100 mM NaCl; 2 mM DTT; 0.01% Brij 35) for 2 hours at 30 °C. Isolated beads bearing cortactin-GFP were then incubated overnight with lysates from Caco-2 cells that had been transfected with cortactin siRNA (20 nM, lysed 48 hours post-transfection) to reduce potential residual cortactin-SRGAP1 complexes in the lysates. The beads were washed thoroughly and boiled in Western blotting lysis buffer, separated by SDS-PAGE and subjected to Western analysis.

Trypsin protection assay
This assay was performed to check the surface levels of E-cadherin [5]. Caco-2 cells were grown to 100% confluence in 35 mm tissue culture dishes, and incubated at 37 °C for 10 min with 0.01% crystalline trypsin in HBSS containing either 2 mM $CaCl_2$ (Ca^{2+}+) or 2 mM EDTA (Ca^{2+}-) for 30 min. Trypsinized cells were pelleted and lysed in Western blotting lysis buffer and boiled for 10 min. Untreated cells were used as a control for total cellular E-cadherin protein. Equal volume of lysates was resolved by SDS-PAGE. Proteins were transferred to a nitrocellulose membrane and probed using an antibody directed specifically to the extracellular domain of E-cadherin.

Nuclei Tracking
Nuclei in Caco-2 cells were identified with NucBlue (Invitrogen) following the manufacturer's instructions. The nuclei were imaged at a 390 nm excitation and a 440 nm emission on the Nikon Ti-E deconvolution microscope with twelve-minute intervals between frames for six hours in the movie medium. Nuclei in movies were tracked using the u-track software developed by the Danuser lab (http://lccb.hms.harvard.edu/software.html) [6, 7]. After successful tracking of >90% of nuclei, tracks were extracted and plots of XY trajectories and calculations of nuclei mean square displacements $\langle \Delta x^2+\Delta y^2 \rangle$ for different time intervals τ were obtained with a custom made MATLAB script. This script is available upon request. The values from the tracks of dividing cells showed apparent overflows in displacements as these were terminated at the beginning of cell division. These tracks were manually excluded from the analysis.

Two-photon laser ablation and tension measurements
To access junctional tension, E-cad-GFP labelled cell contacts were ablated by irradiation with a two-photon laser [8]. The separation of vertices of the ablated contacts was recorded by time lapsing microscopy. The distance between vertices (L) of the contacts was tracked in ImageJ and measured as a function of time (t). The distance values after ablation were subtracted from the initial contact length [L(0)]. The values of $L(t) – L(0)$ were averaged across the contacts tested in each experiment. These mean values were plotted against time to obtain the initial recoil.

The cell-cell junctions were modeled as a Kelvin-Voigt fiber [9, 10], and their deformation [$L(t) – L(0)$] was described by the following equation:

$$L(t) - L(0) = \frac{F_0}{E} \cdot \left(1 - \mathrm{e}^{-\frac{E}{\mu} \cdot t}\right)$$

where F is the force applied to the junction before ablation, E is the elastic modulus of the junction and μ is the viscosity coefficient related to the viscous drag of the media.

We calculated initial recoil velocity as:

$$\text{Initial recoil} = \frac{\mathrm{d}[L(t) - L(0)]}{\mathrm{d}t} = \frac{F_0}{\mu}$$

where initial recoil velocity reflects the tensile force applied at junctions before the ablation of the junctions, assuming a constant viscosity coefficient among experiments.

We calculated k values to assess changes in elasticity of the junction and/or viscosity of the media as:

$$k = \frac{E}{\mu}$$

FRET measurements
Junctional Rac1 activity was measured by WT/V12/N17-Raichu-Rac FRET biosensors [11]. Junctional RhoA activity was measured by a pTriEx-RhoA FRET biosensors [12]. For SRGAP1 knockdown cells, Caco-2 cells were first transfected with SRGAP1 siRNA (50 nM) and 24 hours later transfected with FRET biosensors (1 µg ml^{-1}). The cells were imaged 24 hours after the second transfection on a Zeiss LSM-710 confocal microscope (100X, 1.46NA Plan Apo objective) equipped with a Argon laser and 37 °C heating stage. For cortactin knockdown cells, Caco-2 cells were transfected with cortactin siRNA (50 nM) with the FRET biosensor (1 µg ml^{-1}). The cells were imaged 36 hours after the transfection. The Donor (CFP) and FRET channels were excited using the 458 nm laser line and emissions were recorded between 470 and 490 nm

(Donor) and between 530 and 590 nm (Acceptor, YFP), respectively. The Acceptor channel was excited using the 514 nm laser line and the emission was recorded between 530 and 590 nm.

For FRET analysis the average FRET/CFP emission ratios were calculated on a pixel-by-pixel basis at the apical junctions [13], [14]. The apical junctions were selected as regions of interests (ROIs) for analysis using the freehand tool in ImageJ. The linear pixels within the ROIs of YFP and CFP channels were determined before FRET calculation as described below. Only the pixels in the FRET channel correspondent to the pixels identified to be linear in both YFP and CFP channels were analysed for FRET ratios. The FRET/CFP ratio of a pixel was calculated from the intensity value of a pixel from the FRET channel and a correspondent pixel value from the CFP channel. The average FRET/ CFP ratio of junctional pixels was calculated in an image and mean ratios across 20 images were calculated in each experiment. Mean ratios of three independent experiments were plotted and used for statistical analysis.

To determine the linear pixels in our images, we calculated FRET/CFP and FRET/YFP emission ratios for every pixel using all of the pixels present within the ROIs across all of the images of the control condition. Then all of the pixels were sorted according to Acceptor and Donor intensity values. For each intensity value, an average FRET emission ratio was calculated using a custom made MATLAB script. The average FRET emission ratios for FRET/CFP and FRET/YFP were plotted against Donor and Acceptor intensities, respectively. Non-linear behaviour was seen as an overflow of the FRET emission ratio (FRET/CFP or FRET/YFP), and an appropriate threshold of intensity values was determined to exclude the pixels within this non-linear region before further analysis.

Cell-substrate Adhesion Assay

Cell-substrate adhesion was measured as previously described with modifications [5]. Nitrocellulose-coated 6-well plates were incubated with fibronectin [10 µg ml^{-1} in phosphate-buffered saline (PBS)] overnight at 37 °C. Caco-2 cells were isolated by 0.01% (w/v) crystalline trypsin (Sigma) in HBSS containing 5 mM $CaCl_2$. For each condition of an experiment, freshly isolated cells were allowed to attach to the substrata of a 6-well plate at 37 °C in a CO_2 incubator for 3 hours. Three wells of the 6-well plate were subjected to detachment by systematic pipetting. Five regions of each well (the centre and four quadrants) were flushed with 100 µl PBS drops twice using stand-mounted pipette. After brief PBS washing, the remaining adherent cells were incubated with 200 µl 3-(4,5-dimethylthiazol-2-yl)-2,5-diphenyltetrazolium bromide (MTT, 10 mg ml^{-1} in Me_2SO) and 800 µl PBS at 37 °C for 2 hours. The MTT was removed, and the cells were collected with 200 µl Me_2SO and read at A_{595}. The readouts from the wells subjected to detachment were normalized to the mean readout of the three wells without detachment. The ratios were calculated as the result of the experiment. Cortactin knockdown and reconstitutions were introduced into the cells by lentiviral transduction. For SRGAP1 knockdown, the cells were transfected with SRGAP1 siRNA (100 nM) 48 hours before the assay. For HGF treatment (50 ng ml^{-1}), the cells were treated for 13 hours before the start of the assay and during the 3 hours attachment.

Modelling of RhoA regulation by SRGAP1 and NMIIA

To assess how SRGAP1 contributes to the downregulation of adherens junctions and junctional contractility, we extended our previous computational model to analyse the role of SRGAP1 in RhoA regulation at junctions.

As we previously described, the model employed a set of pairwise stimulation or repression relationships that modulate the steady-state concentration of active species (i.e. molecules that can engage downstream signalling) at junctions using standard Hill function (θ) activation and repression rate equations [15, 16]. Given the components X and Y, where X stimulates or represses Y, the rate of change of Y at the junctional cortex is given by the equations

$$\frac{dY}{dt} = -aY + b\theta_X^Y \quad \text{(for stimulation)}$$

or

$$\frac{dY}{dt} = -aY + b\left(1 - \theta_X^Y\right) \quad \text{(for repression)}$$

where

$$\theta_X^Y = f\left(X, K_X^Y\right) = \frac{X^n}{(K_X^Y)^n + X^n}$$

While interactions may be complex when multiple components act on a single molecule, broadly speaking the constant **a** in the model is the rate at which Y is lost from cell junctions, the constant **b** is the maximal rate of production of Y at the cortex and the constant $\mathbf{K_X^Y}$ corresponds to a dissociation constant between X and Y in the original Hill model. $\mathbf{K_X^Y}$ can be also interpreted as the junctional concentration of X at which the Hill function $\boldsymbol{\theta}$ has half its maximum value.

As noted previously [1], this model deals only with the junctional content of active molecules and how this modulates the cortical accumulation (i.e. stimulation or repression) of downstream effectors, based on the following experimental observations:

1. Inhibition of RhoA by treatment with C3-T decreases junctional amounts of RhoA and its effector ROCK1 [1].
2. Junctional RhoA (and GTP-RhoA, monitored with GFP-AHPH) are depleted when p190B localizes to the junctions [1].
3. Rnd3 junctional localization increases when myosin or ROCK are inhibited [1].
4. Junctional Rnd3 is necessary for p190B RhoGAP (p190B) to localize to the junctions when myosin or ROCK are inhibited [1].
5. Blocking ROCK activity reduces junctional Myosin IIA [17].
6. Junctional Myosin IIA is necessary to localize ROCK1 to the junctions [1].
7. Junctional SRGAP1 is increased when Cortactin 3YF is expressed, and decreased by expression of cortactin 3YD. Further, increased SRGAP1 downregulates junctional RhoA signaling but not Rac1 or Cdc42 (this study).
8. Junctional SRGAP1 is increased in cells treated with HGF via a pathway that requires tyrosine-dephosphorylation of cortactin (this study).
9. Downregulation of junctional RhoA and its activity depend on the GAP domain of SRGAP1 (this study).

Using this approach, we modelled the system in Figure 5f, which results in seven first order differential equations that describe the rates of change in the net concentration at the junctions of signalling capable RhoA, ROCK1, p190B, Rnd3 and NMIIA (Supplementary Table 2; i.e. inactive species are not considered explicitly within this model). Since SRGAP1 and its GAP domain (Fig 4) are required for downregulation of junctional RhoA (and junctional RhoA activity) without affecting RhoA total protein levels and/or Rac1/Cdc42 activity, we tested in the model the effect of SRGAP1 on steady-state junctional RhoA/ROCK1 and NMIIA, by increasing linearly its junctional concentration over time according to:

$$\frac{\mathrm{dSRGAP1}}{\mathrm{dt}} = 0.0075$$

Thus, in our model, increasing the junctional concentration of SRGAP1 implicitly increases total junctional RhoA GAP activity –without increasing SRGAP1 intrinsic GAP activity-, since it causes loss of junctional RhoA (Equation 5, Supplementary Table 2). We thus emphasize that our analysis considers the net signaling of populations of molecules at the junctions, rather than regulation of their intrinsic catalytic activity.

MATLAB was used to solve the system numerically and obtain time courses for the components using the values for the constants and initial conditions shown in Supplementary Table 2.

Supplementary References

1. Priya, R. *et al.* Feedback regulation through myosin II confers robustness on RhoA signalling at E-cadherin junctions. *Nat Cell Biol* **17**, 1282-1293 (2015).
2. Strubbe, G. *et al.* Polycomb purification by in vivo biotinylation tagging reveals cohesin and Trithorax group proteins as interaction partners. *Proc Natl Acad Sci U S A* **108**, 5572-5577 (2011).
3. Ruelcke, J.E., Loo, D. & Hill, M.M. Reducing the cost of semi-automated in-gel tryptic digestion and GeLC sample preparation for high-throughput proteomics. *J Proteomics* **149**, 3-6 (2016).
4. Pinder, A. *et al.* JIP4 is a PLK1 binding protein that regulates p38MAPK activity in G2 phase. *Cell Signal* **27**, 2296-2303 (2015).
5. Verma, S. *et al.* Arp2/3 activity is necessary for efficient formation of E-cadherin adhesive contacts. *The Journal of biological chemistry* **279**, 34062-34070 (2004).
6. Jaqaman, K. *et al.* Robust single-particle tracking in live-cell time-lapse sequences. *Nat Methods* **5**, 695-702 (2008).
7. Ng, M.R., Besser, A., Danuser, G. & Brugge, J.S. Substrate stiffness regulates cadherin-dependent collective migration through myosin-II contractility. *J Cell Biol* **199**, 545-563 (2012).
8. Liang, X., Michael, M. & Gomez, G.A. Measurement of Mechanical Tension at Cell-cell Junctions Using Two-photon Laser Ablation. *Bio Protoc* **6** (2016).
9. Fernandez-Gonzalez, R., Simoes Sde, M., Roper, J.C., Eaton, S. & Zallen, J.A. Myosin II dynamics are regulated by tension in intercalating cells. *Dev Cell* **17**, 736-743 (2009).
10. Michael, M. *et al.* Coronin 1B Reorganizes the Architecture of F-Actin Networks for Contractility at Steady-State and Apoptotic Adherens Junctions. *Dev Cell* **37**, 58-71 (2016).
11. Itoh, R.E. *et al.* Activation of rac and cdc42 video imaged by fluorescent resonance energy transfer-

based single-molecule probes in the membrane of living cells. *Mol Cell Biol* **22**, 6582-6591 (2002).
12. Pertz, O., Hodgson, L., Klemke, R.L. & Hahn, K.M. Spatiotemporal dynamics of RhoA activity in migrating cells. *Nature* **440**, 1069-1072 (2006).
13. Acharya, B.R. *et al.* Mammalian Diaphanous 1 Mediates a Pathway for E-cadherin to Stabilize Epithelial Barriers through Junctional Contractility. *Cell Rep* **18**, 2854-2867 (2017).
14. Ratheesh, A. *et al.* Centralspindlin and alpha-catenin regulate Rho signalling at the epithelial zonula adherens. *Nature cell biology* **14**, 818-828 (2012).
15. Alon, U. *An Introduction to Systems Biology: Design Principles of Biological Circuits*. ((Chapman & Hall/CRC, 2006).
16. Brandman, O., Ferrell, J.E., Jr., Li, R. & Meyer, T. Interlinked fast and slow positive feedback loops drive reliable cell decisions. *Science* **310**, 496-498 (2005).
17. Smutny, M. *et al.* Myosin II isoforms identify distinct functional modules that support integrity of the epithelial zonula adherens. *Nature cell biology* **12**, 696-702 (2010).
